# Editorial: Perspectives and opinions in health services: 2022

**DOI:** 10.3389/frhs.2023.1295810

**Published:** 2023-10-13

**Authors:** Andrea Cioffi, Daniel Ślęzak, Marlena Robakowska, Prestige Tatenda Makanga, Farshid Aleaddini, Camilla Cecannecchia

**Affiliations:** ^1^ Department of Clinical and Experimental Medicine, University of Foggia, Foggia, Italy; ^2^ Division of Medical Rescue, Medical University of Gdansk, Gdansk, Poland; ^3^ Department of Surveying and Geomatics, Midlands State University, Gweru, Zimbabwe; ^4^ Tehran Heart Center, Tehran University of Medical Sciences, Tehran, Iran; ^5^ Department of Anatomical, Histological, Forensic and Orthopedic Sciences, Sapienza University, Rome, Italy

**Keywords:** health, quality, health facility, patient safety, risk management

**Editorial on the Research Topic** Perspectives and opinions in health services: 2022

## Introduction

Knowing what affects the effectiveness and efficiency of health services is the first necessary step to aim. Firstly, the quality of health services is the result of the *a priori* implementation of interventions considering extrinsic variables that affect their applicability, even in the same territory. But not only that, it is based on a critical review of the internal limits of specific health and academic institutions, in the perspective of the noble objective of protect public health.

Our Research Topic saw the publication of 12 papers all valid points of reflection on the topic.

### Proposals to optimize the organization of health systems and to improve the training of health professionals

Zou started from the definition of the world as a “global village”, heterogeneous in ethnicity and sexual orientation, that inevitably results in an increasingly diverse patient population. Language, culture, and gender identity can have a crucial impact on the patient’s health experience. Hence the need to develop a competent and equally diverse workforce in the health sector, able to guarantee fair healthcare.

On a similar wavelength, Balak et al. argue that the management of health systems in accordance with the principles of new public management systems and technological advances risks underestimating the ethical implications underlying any decision in the health field. This could negatively affect the training of resident doctors, scientific research and therefore the real and full efficiency of health care. The authors therefore propose to integrate independent ethics committees in administrative decision-making processes.

Nagele, through an original parallelism between leadership challenges of large academic medical centers (AMC) and large army units, proposed a leadership education program for health professionals drawing on the military model. This responds to the need—recognized by many institutions—to provide training to health professionals who often find themselves in leadership roles without having been properly trained for it.

### Proposals to provide health services to disadvantaged populations, vulnerable patients, and health professionals

Marini et al. proposed a model for delivery of volume sweep imaging (VSI) lung teleultrasound. This model, also used during the COVID-19 pandemic in rural Peru, has garnered acclaim from patients and physicians, and attempts to overcome the many limitations of diagnostic imaging in similar territories around the world.

Talarico et al. proposed a methodology for the optimization of patient care pathways in rare and complex diseases (RarERN Path™). The approach—based on the indispensable involvement of different stakeholders (patients’ representatives, healthcare professionals, hospital managers, and experts in a healthcare organisation)—could contribute to delivering concrete health benefits to these patients whose healthcare has been particularly affected by the COVID-19 pandemic.

Gharebaghi et al. highlighted poor health and social protection for health workers in Iraq. During their career, health professionals face a large list of challenges (in the economic, social, and professional spheres) that have been further exacerbated during the COVID-19 period, with inevitable repercussions on mental well-being. These data, together with the numerous suicides recorded in Iraq among the physician residents, make clear the need to provide all healthcare professionals free access to psychiatric counseling for preventive purposes.

### Proposals and opinions for the implementation of interventions in health field

Nilsen et al. through the examination of the objectives and characteristics of the four scientific fields (intervention, innovation, implementation, and improvement sciences), have proposed interesting ideas on the optimization of the use and adaptation of artificial intelligence in the healthcare sector. Indeed, the application of artificial intelligence in healthcare should start from the critical evaluation of its usefulness in the various fields of science.

Pérez Jolles et al. argue that the promotion of collaborative approaches is crucial to achieving synergistic goals in the field of implementation science. The authors proposed a guide based on five principles, useful for researchers to structure implementation collaborations with a variety of stakeholders (co-creation). The effective involvement of partners in the implementation of the services they finance, provide, or receive could help bridge the gap between what we know in theory and the actual implementation of health interventions.

Fort et al. proposed the use of the Practical Implementation Sustainability Model (PRISM) for the implementation of health programs. PRISM, for its structural characteristics (such as multi-level assessments of the characteristics of the intervention, the environment, and the target subjects) could be effectively adapted in the healthcare sector with an equity lens to tackle health inequalities at the root, that is, from the planning and implementation of interventions.

Knox and Curran agreed with researchers who promote the adoption of an effectiveness-implementation hybrid design in the health field, especially in those contexts where implementation must focus on *a priori* assessment of possible barriers to effectiveness. In support of their thesis, the authors presented implementation data that could have been collected if vaccine efficacy trials used hybrid designs and that would have allowed to predict the vaccination hesitation.

In a similar vein, Leeman et al. proposed a method to identify how external influences can impact the implementation of new healthcare interventions. The authors formulated 20 propositions from five classic organization theories (Complexity, Contingency, Institutional, Resource Dependence, and Transaction Cost Economics theory) which they used for the implementation of intervention for the prevention of tobacco smoke. The classical theories of the organization can be a useful empirical support to develop implementation strategies and to understand external factors that can influence them.

Kalver et al. proposed a novel consensus group approach—the CORE (Consensus on Relevant Elements) approach—to determine the initial core components of the Department of Veterans Affairs (VA)’s Post-Incarceration Engagement (PIE) program, to date implemented in only two states in the United States but in increasing diffusion. The Core approach is a multi-step process that involves a team of experts and moderators and can be a guide to determine the initial core components, to be understood as the principles and essential elements of evidence-based interventions. The systematic isolation of core components is fundamental to allow the health interventions to be improved and adapted to other application contexts.

## Conclusion

Despite the various issues addressed, the common thread between these papers is that improving the effectiveness and efficiency of health services can mean, at the same time, drawing on classic reference models and innovative tools. Both, however, should be balanced and adapted to changing external factors and to ethical implications underlying any intervention in the health field.

